# *In Silico* Investigation of Potential Pyruvate Kinase M2 Regulators from Traditional Chinese Medicine against Cancers

**DOI:** 10.1155/2014/189495

**Published:** 2014-06-25

**Authors:** Kuan-Chung Chen, Kuen-Bao Chen, Hsin-Yi Chen, Calvin Yu-Chian Chen

**Affiliations:** ^1^School of Pharmacy, China Medical University, Taichung 40402, Taiwan; ^2^School of Medicine, College of Medicine, China Medical University, Taichung 40402, Taiwan; ^3^Department of Biomedical Informatics, Asia University, Taichung 41354, Taiwan; ^4^Department of Anesthesiology, China Medical University Hospital, Taichung 40447, Taiwan; ^5^Human Genetic Center, Department of Medical Research, China Medical University Hospital, Taichung, Taiwan; ^6^Research Center for Chinese Medicine & Acupuncture, China Medical University, Taichung 40402, Taiwan

## Abstract

A recent research in cancer research demonstrates that tumor-specific pyruvate kinase M2 (PKM2) plays an important role in chromosome segregation and mitosis progression of tumor cells. To improve the drug development of TCM compounds, we aim to identify potent TCM compounds as lead compounds of PKM2 regulators. PONDR-Fit protocol was utilized to predict the disordered disposition in the binding domain of PKM2 protein before virtual screening as the disordered structure in the protein may cause the side effect and downregulation of the possibility of ligand to bind with target protein. MD simulation was performed to validate the stability of interactions between PKM2 proteins and each ligand after virtual screening. The top TCM compounds, saussureamine C and precatorine, extracted from *Lycium chinense* Mill. and *Abrus precatorius* L., respectively, have higher binding affinities with target protein in docking simulation than control. They have stable H-bonds with residues A:Lys311 and some other residues in both chains of PKM2 protein. Hence, we propose the TCM compounds, saussureamine C and precatorine, as potential candidates as lead compounds for further study in drug development process with the PKM2 protein against cancer.

## 1. Introduction

Recently, more and more pathogenesis of diseases has been identified [1, 2] to reveal potential target proteins for drug design [3–5]. A recent research in cancer research demonstrates that tumor-specific pyruvate kinase M2 (PKM2) plays an important role in chromosome segregation and mitosis progression of tumor cells [6, 7]. PKM2 proteins can be treated as drug target proteins against cancers [8, 9].

The computer-aided drug design had wildly been used for virtual drug screening in the drug design [10, 11]. In previous study, many compounds from traditional Chinese medicine (TCM) have been identified as potential lead compounds in computer-aided drug design for the treatment of cancers [12–14], metabolic syndrome [15], diabetes [16], stroke [17, 18], inflammation [19], and some other diseases [20]. To improve the drug development of TCM compounds, we employed TCM compounds from TCM Database@Taiwan [21] to virtual screening of the potent lead compounds of PKM2 regulators. As the disordered structure in the protein may cause the side effect and downregulation of the possibility of ligand to bind with target protein [22], PONDR-Fit protocol was performed to predict the disordered disposition in binding domain of PKM2 protein before virtual screening. The MD simulation was performed after virtual screening, to validate the stability of interactions between PKM2 proteins and each ligand in each docking pose.

## 2. Materials and Methods

### 2.1. Data Collection

The X-ray crystallography structure of the human pyruvate kinase M2 (PKM2) was downloaded from RCSB Protein Data Bank with PDB ID: 4G1N [9]. To predict the disordered disposition in PKM2 protein, PONDR-Fit [23] protocol was employed with the sequence of PKM2 protein from Swiss-Prot (UniProtKB: P14618). In preparation section, the final structure of PKM2 protein was protonated with Chemistry at HARvard Macromolecular Mechanics (CHARMM) force field [24] and removed water molecules in the X-ray crystallography structure by Prepare Protein module in Discovery Studio 2.5 (DS2.5). The TCM compounds from TCM Database@Taiwan [21] were filtered by Lipinski's Rule of Five [25], and their final structure was protonated using Prepare Ligand module in DS2.5. The binding site was defined by the volume of the cocrystallized PKM2 activator for virtual screening.

### 2.2. Docking Simulation

The TCM compounds were docking into the binding site defined above by LigandFit protocol [26] in DS 2.5 using a shape filter and Monte-Carlo ligand conformation generation. CHARMM force field [24] was employed to optionally minimize the docking poses, and then the clustering algorithm was employed to filter out the similar poses. Each docking pose was evaluated by Dock Score using the following equation:

(1) Dock  Score=−(ligand  receptor  interaction  energy+ligand  internal  energy).

### 2.3. Molecular Dynamics (MD) Simulation

The molecular dynamics (MD) simulation was employed with classical molecular dynamics theory using Gromacs 4.5.5 [27] to simulate the variation of each protein-ligand complex under dynamic conditions. In preparation section, the PKM2 proteins were prepared by pdb2gmx protocol of Gromacs to provide topology and parameters with CHARMM27 force field, and each ligand was prepared by SwissParam program [28] to provide topology and parameters with CHARMM. A cubic box solvated using TIP3P water model and 0.145 M NaCl model was defined based upon the edge approx. 12 Å from the protein complexes periphery. In the minimization section, we employed steepest descents [29] minimization with a maximum of 5,000 steps to remove bad van der Waals contacts. In the equilibration section, we perform position-restrained molecular dynamics with the linear constraint algorithm for all bonds by Gromacs program with NVT equilibration, Particle Mesh Ewald method, and Berendsen weak thermal coupling method. In the production section, we perform 10,000 ps production simulation by Gromacs program with time step in unit of 2 fs under NPT ensembles and particle mesh Ewald (PME) option. A series of protocols in Gromacs program was employed to analyze the MD trajectories of 5000 ps.

## 3. Results and Discussion

### 3.1. Disordered Protein Prediction

**Figure 1:**
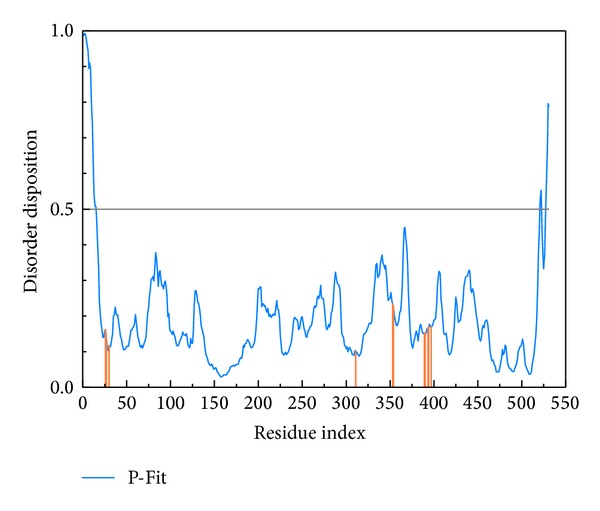
Disordered disposition predicted by PONDR-Fit.

The disordered disposition predicted by PONDR-Fit protocol with the sequence of PKM2 protein from Swiss-Prot (UniProtKB: P14618) was displayed in Figure 1. The key residues in the binding site do not locate in disordered domain (>0.5), which indicates that PKM2 protein expresses a stable binding domain in protein folding. We employed the crystallography structure of PKM2 protein for docking simulation as the residues in the binding site of target protein have no significant variation.

### 3.2. Docking Simulation

**Figure 2:**
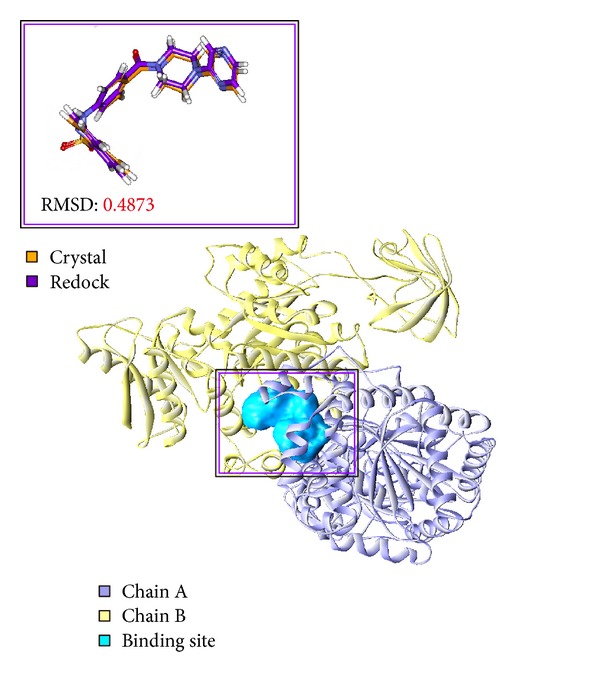
Binding site of PKM2 protein defined as the volume of *N*-(4-((4-(pyrazin-2-yl)piperazin-1-yl)carbonyl)phenyl)quinoline-8-sulfonamide and root-mean-square deviation value between crystallized structure (orange) and docking pose (violet).

**Figure 3:**
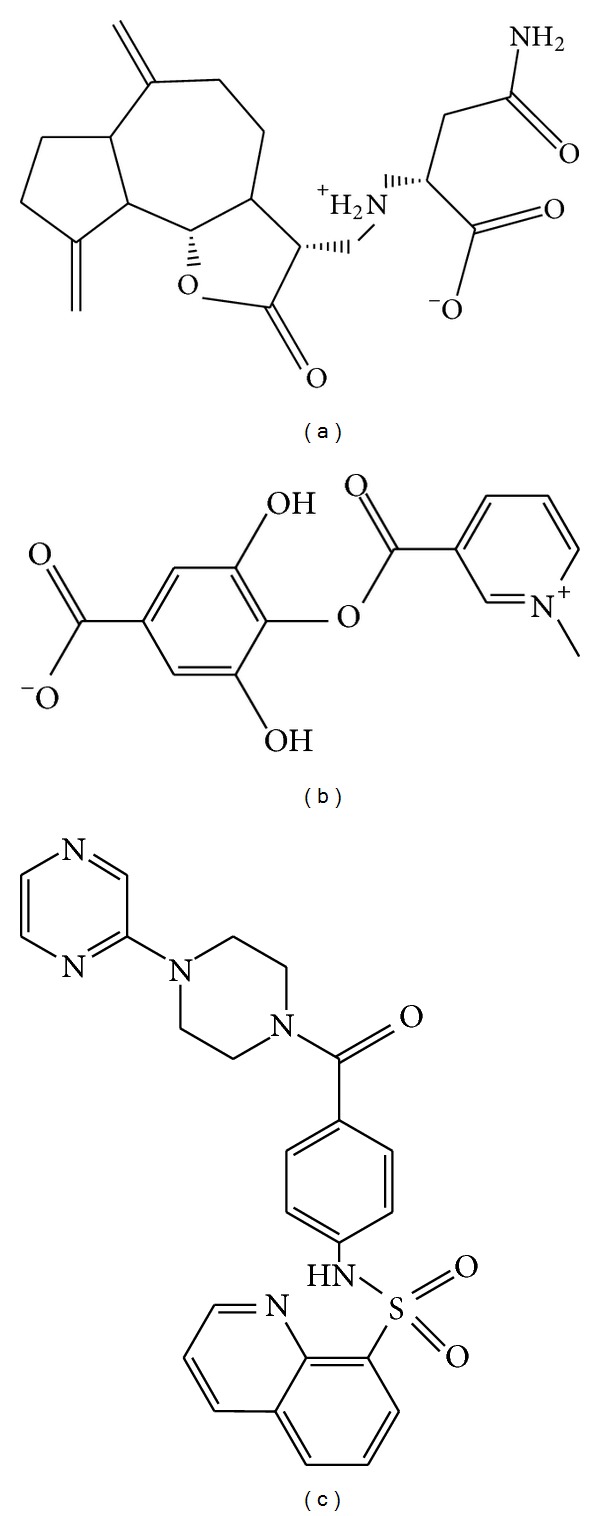
Chemical scaffold of controls and top two TCM candidates with their scoring function and sources. (a) Saussureamine C, (b) precatorine, and (c) NZT.

**Figure 4:**
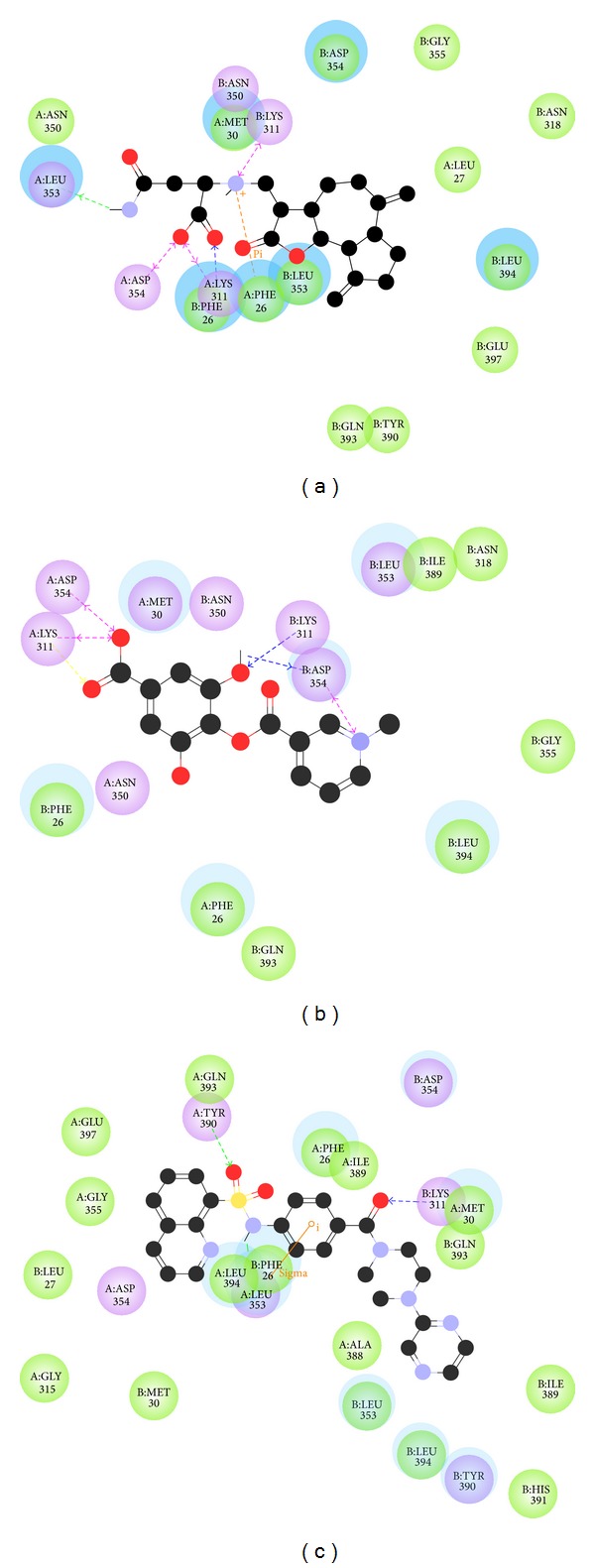
Docking pose of PKM2 protein complex with (a) saussureamine C, (b) precatorine, and (c) NZT.

**Figure 5:**
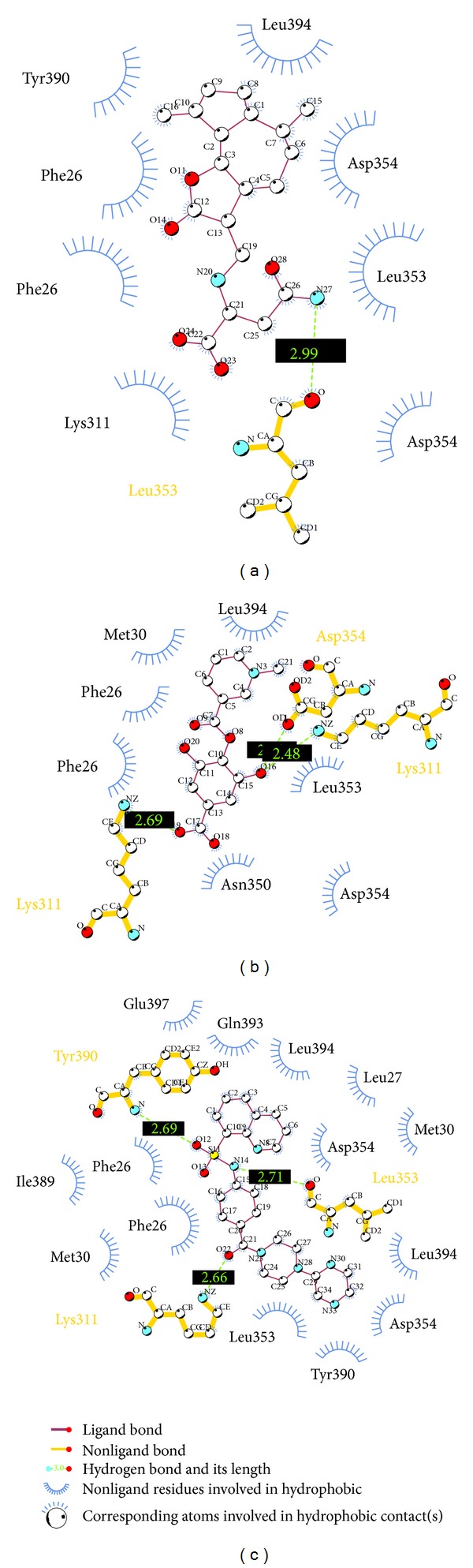
Docking pose of PKM2 protein complex with (a) saussureamine C, (b) precatorine, and (c) NZT drawn by LIGPLOT program.

To validate the accuracy of LigandFit protocol in DS2.5, the cocrystallized PKM2 protein activator was redocked into the binding site of PKM2 protein. The root-mean-square deviation (RMSD) value between crystallized structure and docking pose of control is 0.4873 (Figure 2), which shows a good accuracy in the docking simulation by LigandFit protocol. So we employ LigandFit protocol as suitable for virtual screening with PKM2 protein. The top TCM compounds ranked by Dock Score [26] and control, *N*-(4-((4-(pyrazin-2-yl)piperazin-1-yl)carbonyl)phenyl)quinoline-8-sulfonamide (NZT), are shown in Table 1. For the top two TCM compounds, saussureamine C and precatorine were extracted from* Lycium chinense* Mill. and* Abrus precatorius* L. The chemical scaffold of saussureamine C, precatorine, and control is illustrated in Figure 3. According to the docking poses in Figures 4 and 5, the top two candidate compounds have hydrogen bonds (H-bonds) with common residue B:Lys311 and an interaction with residues in both chains of PKM2 protein as control.

### 3.3. Molecular Dynamics Simulation

**Figure 6:**
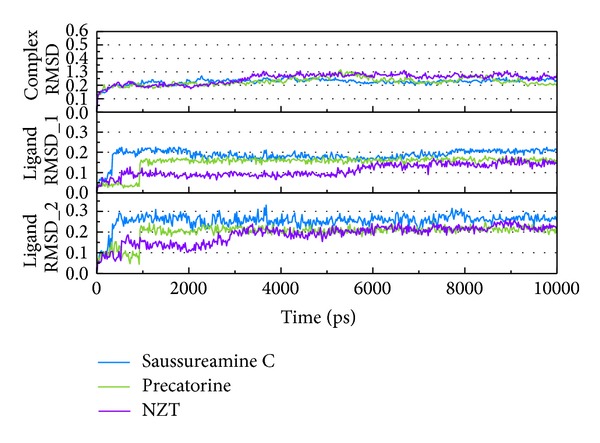
Root-mean-square deviations in units of nm for protein and ligand for PKM2 protein complexes with saussureamine C, precatorine, and NZT. Ligand RMSD_1 and Ligand RMSD_2 are calculated with least squares fit by protein and ligand, respectively.

**Figure 7:**
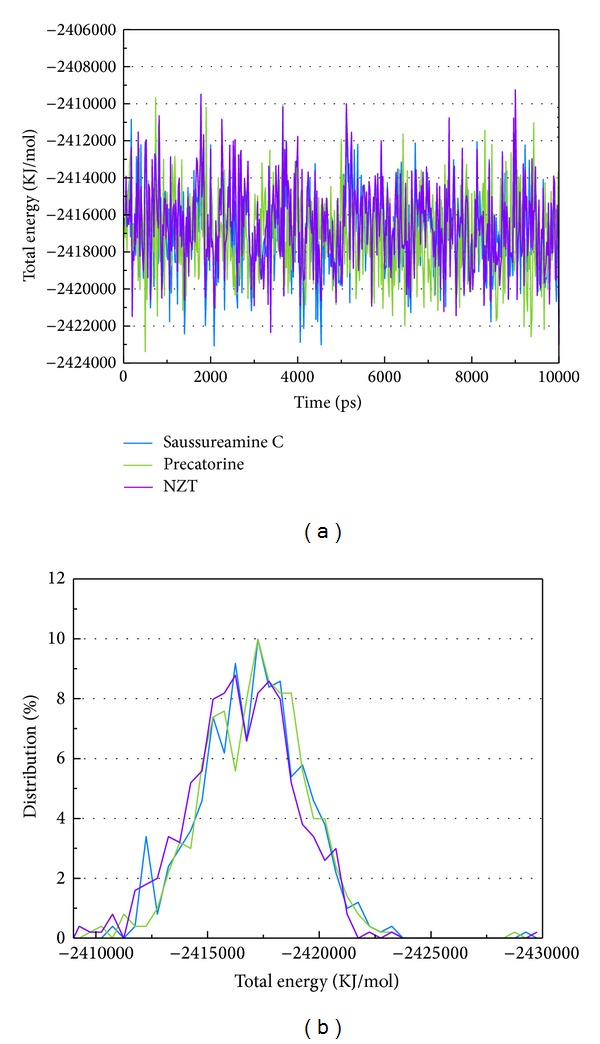
(a) Distribution and (b) variation of total energy for PKM2 protein complexes with saussureamine C, precatorine, and NZT over 5000 ps of MD simulation.

**Figure 8:**
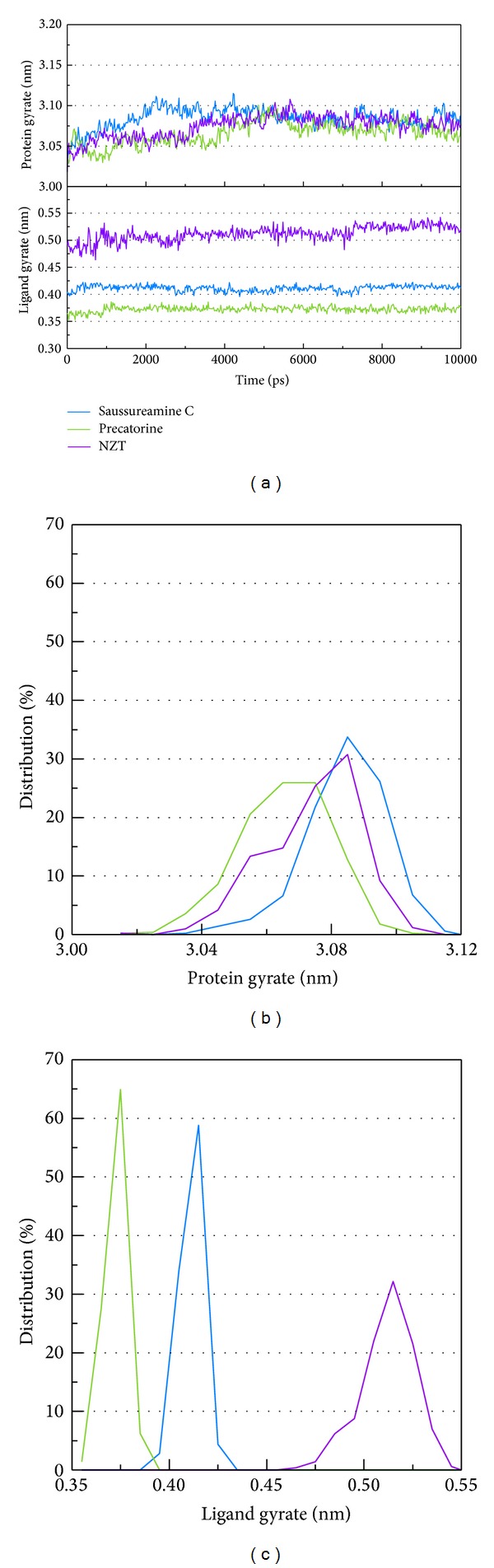
(a) Variation of radii of gyration for protein and ligands in PKM2 complexes with saussureamine C, precatorine, and NZT over 5000 ps of MD simulation. Distribution of radii of gyration for (b) protein and (c) ligands in PKM2 complexes with saussureamine C, precatorine, and NZT over 10,000 ps of MD simulation.

**Figure 9:**
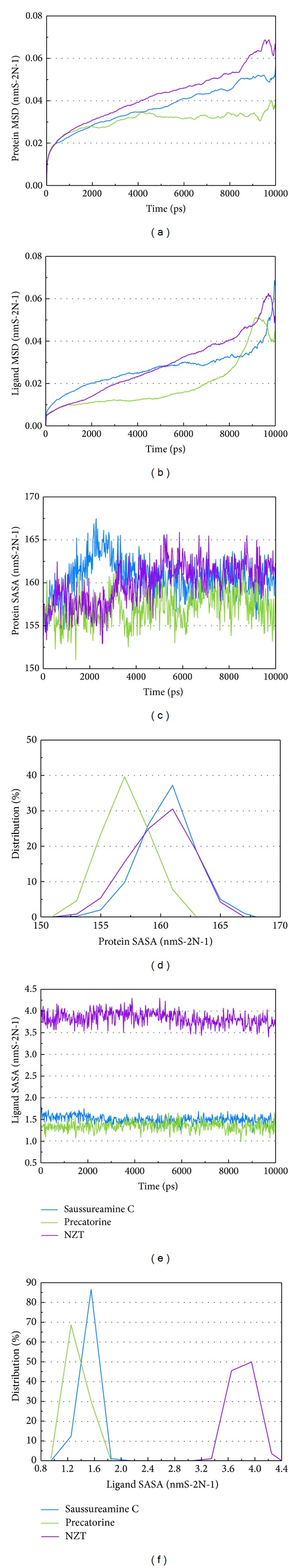
Variation of (a) mean square displacement (MSD) of protein, (b) ligand MSD, (c) total solvent accessible surface area (SASA) of protein, and (e) ligand SASA and distribution of (d) protein SASA and (f) ligand SASA for PKM2 complexes with saussureamine C, precatorine, and NZT over 10,000 ps of MD simulation.

**Figure 10:**
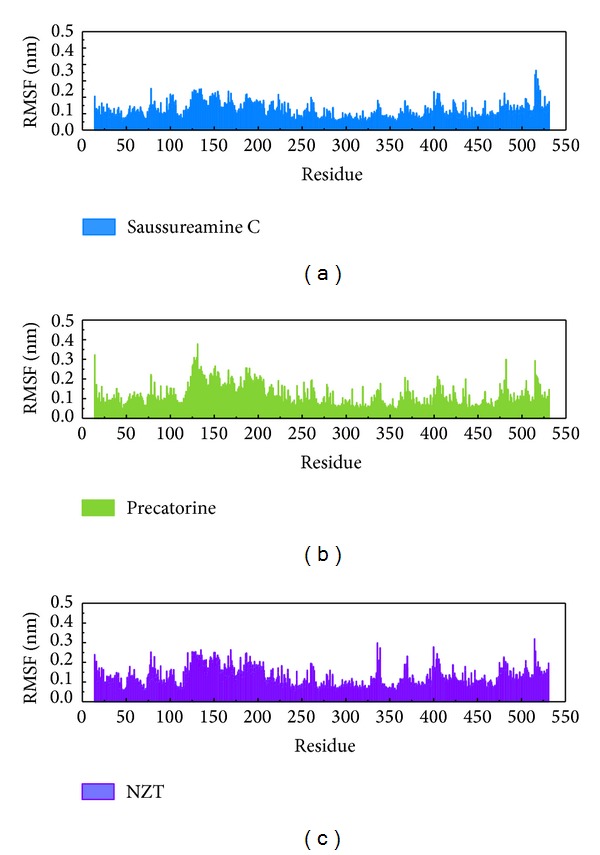
Root-mean-square fluctuation (RMSF) for residues in PKM2 complexes with saussureamine C, precatorine, and NZT.

LigandFit protocol performs a docking simulation with a rigid body of PKM2 proteins. The docking poses with PKM2 protein may modify under dynamic conditions. We employed the MD simulation to validate the stability of interactions of each ligand with PKM2 proteins. Root-mean-square deviations (RMSDs) illustrated the atomic fluctuations during MD simulation.  Figure 6 displays the atomic fluctuations of PKM2 proteins and ligands in complexes with saussureamine C, precatorine, and control during 10,000 ps MD simulation. It shows that PKM2 proteins tend to be stable after a short period of MD simulation. In addition, there is no significant variance for the total energies of each PKM2 protein complex during MD simulation (Figure 7). The variation and distribution of radii of gyration for protein and ligand over 10,000 ps MD simulation in Figure 8 indicate that the radii of gyration of PKM2 protein complexes with ligands, saussureamine C, precatorine, and control were stabilized under dynamics condition after 5,000 ps MD simulation. The variation of mean-square displacement (MSD) and total solvent accessible surface area (SASA) for each protein and ligand displayed in Figure 9. They indicate that the SASA of PKM2 protein in complexes with precatorine was decreased after MD simulation, which implies that precatorine may cause two chains of PKM2 protein more compact. Root-mean-square fluctuations (RMSFs) for each residue over 10,000 ps MD simulation are shown in Figure 10. They indicate that PKM2 proteins docking with saussureamine C and precatorine cause flexibility for PKM2 proteins as control.

**Figure 11:**
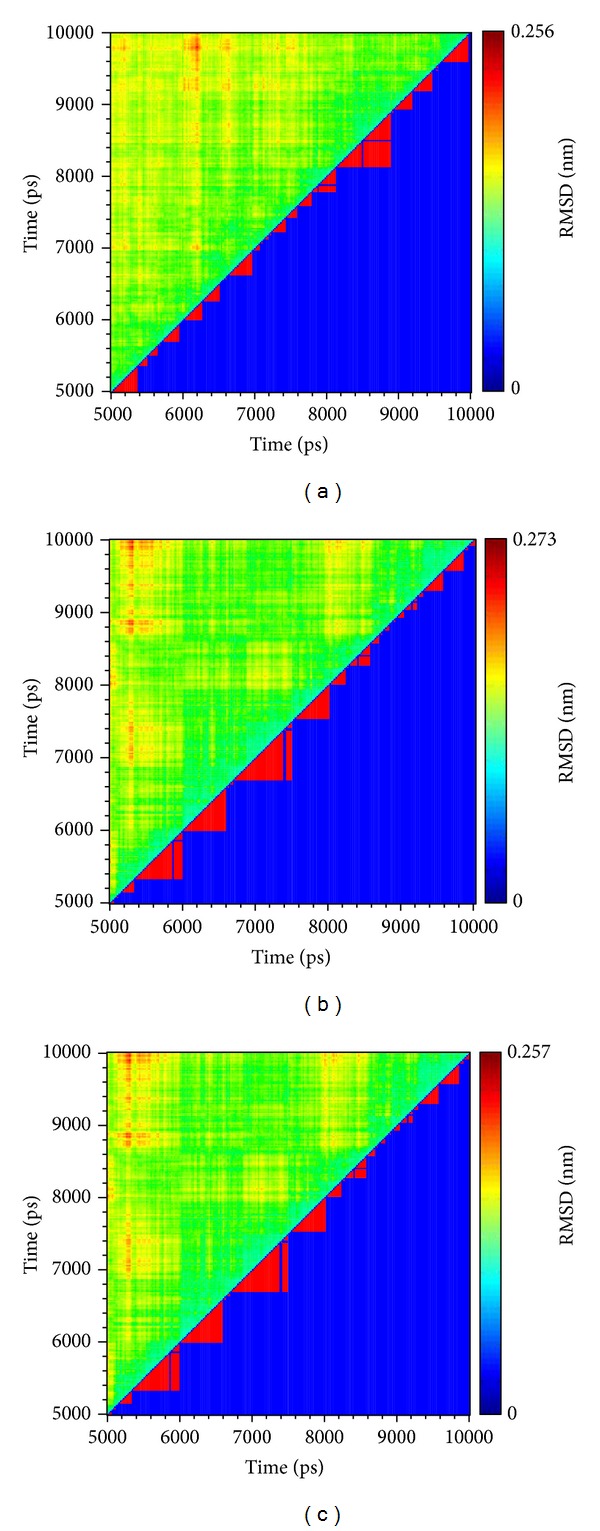
Root-mean-square deviation value (upper left half) and graphical depiction of the clusters with cutoff 0.1 nm (lower right half) for PKM2 complexes with (a) saussureamine C, (b) precatorine, and (c) NZT.

**Figure 12:**
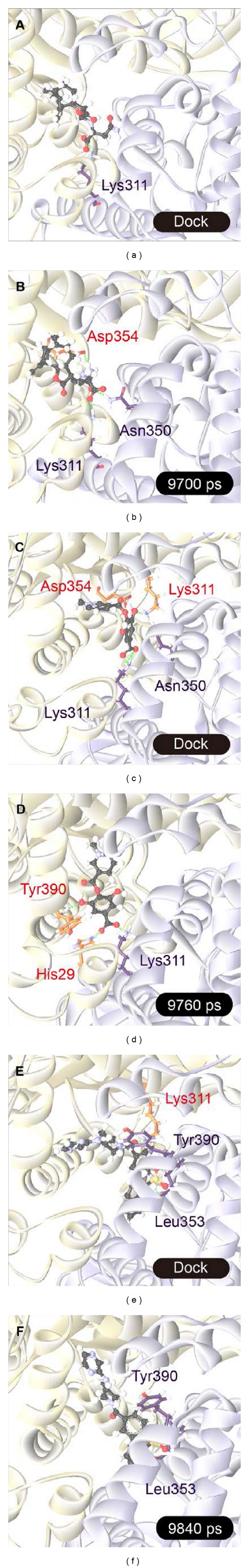
Docking poses in docking simulation (left) and middle RMSD structure in the major cluster (right) for PKM2 complexes with (a), (b) saussureamine C, (c), (d) precatorine, and (e), (f) NZT.

**Figure 13:**
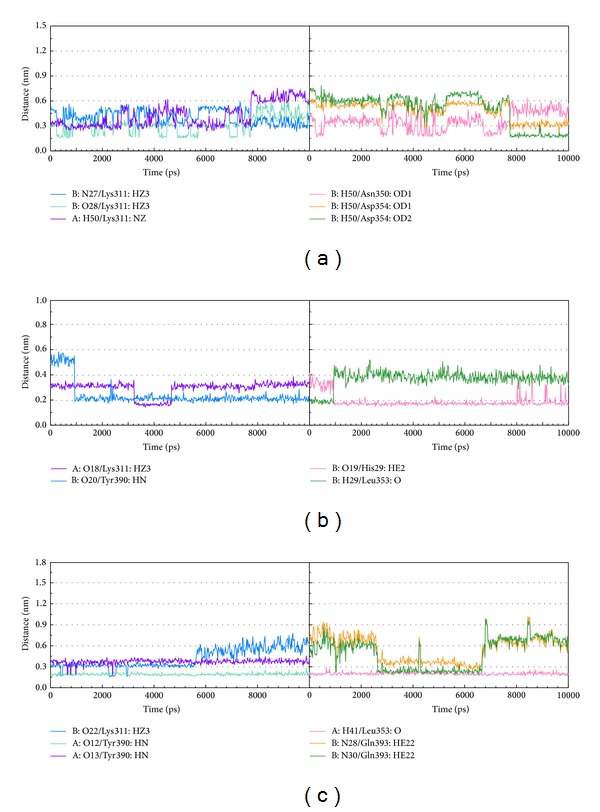
Distance variation of H-bonds with PKM2 protein during MD simulation. (a) Saussureamine C, (b) precatorine, and (c) NZT.

After MD simulation, we decide the representative structures of PKM2 protein complexes by the RMSD values and graphical depiction of the clusters analysis with cutoff of 0.1 nm (Figure 11). To compare with the interactions in docking simulation and in representative structures of PKM2 protein complexes after MD simulation, the snapshots of each docking pose were displayed in Figure 12. They indicate that two TCM candidates remain the H-bonds with residues Lys311 in chain A of PKM2 protein.  Table 2 and Figure 13 display the H-bond occupancy and distance variation for each ligand with PKM2 proteins. They indicate that both TCM compounds have stable H-bonds with residues A:Lys311 and some other residues in both chains of PKM2 protein, which can stabilize the docking poses in the binding domain.

## 4. Conclusion

This study aims to investigate the potent lead drug from TCM compounds for PKM2 protein inhibitors against cancers. The top TCM compounds, saussureamine C and precatorine, have higher binding affinities with PKM2 proteins in docking simulation than control. They have H-bonds with residues A:Lys311 and some other residues in both chains of PKM2 protein. After MD simulation, the top TCM compounds maintain the similar docking poses under dynamic conditions. In addition, the top two TCM compounds, saussureamine C and precatorine, were extracted from* Lycium chinense* Mill. and* Abrus precatorius* L., respectively. Hence, we propose the TCM compounds, saussureamine C and precatorine, as potential candidates as lead compounds for further study in drug development process with the PKM2 protein against cancer.

## Figures and Tables

**Table 1:** Scoring functions of top candidates from TCM database screening.

Name	Source	Dock Score
Saussureamine C	*Lycium chinense* Mill.	166.382
Precatorine	*Abrus precatorius *L.	161.002
NZT∗		73.062

*Control:  *N*-(4-((4-(pyrazin-2-yl)piperazin-1-yl)carbonyl)phenyl)quinoline-8-sulfonamide.

**Table 2:** H-bond occupancy for key residues of PKM2 protein with top TCM compounds overall 10,000 ps of molecular dynamics simulation.

Ligand	H-bond	Ligand atoms	Amino acid	Distance (nm)			Occupancy (%)
				Max.	Min.	Average	
Saussureamine C	1	O23	A:Lys311:HZ3	0.553	0.259	0.438	15.80%
	2	O24	A:Lys311:HZ3	0.57	0.146	0.294	94.80%
	3	N27	A:Lys311:HZ3	0.593	0.234	0.408	30.00%
	4	O28	B:Lys311:HZ3	0.726	0.161	0.519	10.40%
	5	O28	B:Lys311:HZ3	0.63	0.15	0.31	73.20%
	6	H50	B:Lys311:NZ	0.76	0.24	0.43	46.20%
	7	H50	A:Asn350:OD1	0.64	0.16	0.36	44.80%
	8	H52	B:Asp354:OD1	0.54	0.27	0.31	96.20%
	9	H50	B:Asp354:OD1	0.66	0.19	0.49	20.40%
	10	H50	B:Asp354:OD2	0.79	0.15	0.51	23.20%

Precatorine	1	O18	A:Lys311:HZ3	0.39	0.14	0.29	98%
	2	O19	B:His29:HE2	0.41	0.15	0.19	97%
	3	O20	B:Tyr390:HN	0.58	0.17	0.24	91%
	4	H29	B:Leu353:O	0.52	0.16	0.37	25%

NZT∗	1	O12	A:Tyr390:HN	0.32	0.16	0.19	100%
	2	O13	A:Tyr390:HN	0.46	0.17	0.37	15%
	3	O22	B:Lys311:HZ3	0.78	0.15	0.42	53%
	4	N28	B:Gln393:HE22	1.03	0.23	0.56	18%
	5	N30	B:Gln393:HE22	0.99	0.19	0.49	40%
	6	H41	A:Leu353:O	0.30	0.16	0.20	100%

H-bond occupancy cutoff: 0.35 nm.

∗Control: *N*-(4-((4-(pyrazin-2-yl)piperazin-1-yl)carbonyl)phenyl)quinoline-8-sulfonamide.
